# Oral-Gut Microbiome Axis in the Pathogenesis of Cancer Treatment-Induced Oral Mucositis

**DOI:** 10.3389/froh.2022.881949

**Published:** 2022-03-28

**Authors:** Ghanyah Al-Qadami, Ysabella Van Sebille, Joanne Bowen, Hannah Wardill

**Affiliations:** ^1^School of Biomedicine, University of Adelaide, Adelaide, SA, Australia; ^2^UniSA Online, University of South Australia, Adelaide, SA, Australia; ^3^Precision Medicine Theme (Cancer), South Australian Health and Medical Research Institute, Adelaide, SA, Australia

**Keywords:** oral mucositis, chemotherapy, radiotherapy, HSCT, oral microbiota, gut microbiota, oral-gut microbiome axis

## Abstract

Oral mucositis (OM) is one of the most common and debilitating oral complications of cancer treatments including chemotherapy, radiotherapy, and hematopoietic stem cell transplantation. It is associated with severe pain and difficulties in chewing, swallowing, and speech. This leads to impairment of basic oral functions and could result in unplanned treatment interruption or modification. As such, OM negatively impacts both patients' quality of life as well as tumor prognostic outcomes. Understanding pathways underlying OM pathogenesis help identify new targets for intervention or prevention. The pathophysiology of OM has been widely studied over past decades with several pathways related to oxidative stress, inflammation, and molecular and cellular signaling being implicated. In this mini-review, we will discuss the emerging role of the oral-gut microbiome axis in the development of OM. Particularly, we will elaborate on how the alterations in the oral and gut microbiota as well as intestinal dysfunction caused by cancer treatments could contribute to the pathogenesis of OM. Further, we will briefly discuss the potential methods for targeting the oral-gut microbiome axis to improve OM outcomes.

## Introduction

The oral/oropharyngeal mucosa is highly sensitive to cytotoxic anti-cancer agents causing profound inflammation and breakdown of the mucosal barrier [1]. The resulting ulcerative lesions, termed oral mucositis (OM), is one of the most frequent oral complications affecting 80–100% of patients with head and neck cancer (HNC) treated with radiotherapy [2, 3], up to 40% of patients receiving chemotherapy [4], and 70–87% of patients undergoing hematopoietic stem cell transplantation (HSCT) [5, 6]. OM has been identified as one of the most debilitating toxicities that significantly impact patients' quality of life due to its associated pain, difficulty chewing and swallowing, weight loss, and infection [2, 7–9]. In cases where these cannot be optimally managed, treatment is often withheld or the dose reduced, which therefore negatively impacts patient prognosis [10, 11]. In addition to clinical consequences, OM is also associated with a significant economic cost as patients often require intensive medical interventions for symptoms management [2, 7, 12].

OM pathophysiology is a complex multifactorial process involving direct and indirect injury pathways including DNA damage, oxidative stress, inflammatory responses, and bacterial translocation [13]. OM develops through five phases i.e., initiation, signal upregulation and amplification, ulceration, and healing [14–19]. Briefly, exposure to cytotoxic agents initiates epithelial cell death through direct DNA damage and the production of reactive oxygen species causing tissue damage and activating subsequent molecular pathways including nuclear factor kappa-B (NF-κB). This results in the production of pro-inflammatory mediators such as tumor necrosis factor-α, interleukin (IL)-1β, and IL-6 leading to the ulcerative phase in which painful deep ulcers are formed creating a thriving environment for bacterial colonization which in turn exacerbate inflammatory responses. During the healing phase, signals from the submucosa and extracellular matrix stimulate proliferation and differentiation of epithelial cells allowing the restoration of the normal tissue structure [14–19].

The significant pathological changes in the oral cavity have led to the assumption that alterations in the oral microbes following cancer treatments could contribute to OM development. As such, the role of oral microbiota in the pathogenesis of OM has been an area of interest for several decades, with changes in oral microbiota following radiation therapy documented as early as the 1980s [20]. The significant increase in bacterial load in the ulcerated epithelium, and the correlation between bacterial load and OM peak severity [19], has suggested a causal relationship between oral bacteria and OM [19, 21, 22]. Hence, multiple clinical trials have used antimicrobial agents targeting oral bacteria to reduce OM severity; however, these attempts have failed to achieve positive outcomes [23]. This might be due to non-selective targeting of the oral microbiota and a lack of understanding of which specific microbes are contributing to OM. The recent advances in culture-independent microbial detection technologies (e.g., 16S rRNA sequencing) have allowed for extensive characterization of oral microbiota and subsequent investigation of its association with OM [24].

In addition to oral pathology, cancer treatments are also associated with major pathological changes in the lower gastrointestinal tract including intestinal inflammation, and disruption of intestinal barrier integrity and functions [13, 25]. These are often accompanied by changes in the gut microbiota, which serve to exacerbate gastrointestinal dysfunction [26]. In addition to disrupting local gut homeostasis, these changes are thought to impact organ systems at distant sites and therefore have prompted speculation that disruption of intestinal homeostasis could also contribute to OM pathogenesis. This mini-review focuses on the role of oral-gut microbiome axis pathways including oral and gut microbiota dysbiosis, intestinal dysfunction, and gut microbiota oralization in OM pathogenesis and briefly discusses potential methods to target these pathways to prevent or reduce the severity of OM.

## Oral-Gut Microbiome Axis in OM

### Oral Microbiota Dysbiosis and OM

The oral microbiota, a collection of microorganisms residing in the oral cavity, is composed of more than 700 bacterial species representing the second-largest microbial community in the human body after the gut microbiota [27]. Different bacterial populations are found in different oral cavity sites with a distinctive microbial community found in saliva, oral mucosa, and dental plaque [28, 29]. Oral microbiota plays a key role in maintaining oral homeostasis and preventing the colonization of exogenous pathogenic microorganisms [28, 30]. However, disruption of the oral microbial ecosystem could contribute to local and systemic diseases, with a growing body of evidence implicating the oral microbiota with oral diseases (periodontitis, dental caries, and oral cancer) and systemic conditions (colorectal cancer, diabetes, Alzheimer's disease, and cardiovascular diseases) [31].

Exposure to cytotoxic cancer therapies is widely associated with changes in the oral microbiota, *directly* caused by bactericidal or bacteriostatic anti-cancer agents, and *indirectly* through the breakdown of the mucosal lining and alteration of immunological properties of the oral environment [32–35]. Similarly, changes in saliva production and composition, xerostomia, are also associated with microbial changes in the mouth [36, 37]. Alterations in the oral microbiota have been extensively studied using both culture-dependent and independent methods. While culture-based studies confirmed the alterations in oral microbiota following cancer treatments, they failed to demonstrate an association between the oral microbiota and OM severity as the analysis was limited to cultivated microorganisms [32]. The rapid advances in culture-independent molecular and next-generation sequencing techniques have allowed for more efficient detection of low abundance and non-cultivable taxa and helped overcome the detection limitations of culture-based methods [34, 38]. Hence, multiple studies have used these methods to characterize the oral microbiota in patients undergoing cancer treatments [39–41]. For instance, using 16S rRNA sequencing, Napeñas et al. reported a shift in the oral microbial community, which was dominated by *Streptococcus mitis* and *Gemella haemolysans* in patients with breast cancer treated with chemotherapy [39]. The same method was used by Hu et al. and demonstrated a temporal shift in the relative abundance of core oral microbiota throughout radiotherapy with a negative correlation between radiation doses and the oral microbial richness in patients with HNC undergoing radiotherapy [40]. Studies also attempted to identify a specific oral microbial signature associated with the risk or severity of OM (Table 1) [6, 33, 41–50]. Although no clear microbial signature was identified across these studies, one of the consistent observations is the enrichment of oral pathobiont *Fusobacterium* (*F. nucleatum*) in patients with severe OM [6, 41, 44–46, 48]. Interestingly, patients who experienced more severe OM had more profound changes in the oral microbiota while a more resilient oral microbiota, minimal alterations, and faster recovery of the microbial community were observed in those with less severe OM [33, 46, 49, 50]. Collectively, the current evidence suggests that oral microbiota alterations are associated with OM onset and severity; however, a clear microbial pattern is yet to be established. This might be due to the variation in study subjects, samples collection time, sampling sites and methodology, or OM scoring methods. Thus, there is a need for a standardized methodology for oral microbiota sampling and analysis to obtain more consistent results.

**Table 1:** Studies investigated the association between the oral microbiota and the development of OM (*studies that used culture-independent methods only were included*).

**Study**	**Subjects**	**Therapy**	**Sampling/analysis method**	**Key findings**
Laheij et al. [6]	Adult patients with hematological malignancies (*n* = 49)	Myeloablative or reduced intensity-conditioning + HSCT	Oral rinsing samples/real-time PCR	The presence and load of *P. gingivalis* were associated with a higher risk of ulcerative OM in non-keratinized and keratinized oral mucosa Percentage (in relation to total load) of *P. gingivalis, P. micra, F. nucleatum*, and *T. denticola* was associated with ulcerative OM in non-keratinized oral mucosa
Ye et al. [33]	Pediatric patients with hematological and solid malignancies (*n* = 37) Healthy children (*n* = 38)	Chemotherapy	All patients and controls: lip and buccal mucosa samples Patients with mucositis: lesion samples/16S rRNA gene 454 pyrosequencing	Pre-chemotherapy, patients who developed OM had higher microbial diversity and increased abundance of Bacteroidetes (*Capnocytophaga*), Firmicutes (*Peptostreptococcaceae Incertae Sedis, Lactococcus*), *Fusobacteria*, and *Spirochaetes* During chemotherapy, patients who developed OM had more pronounced alterations in bacterial composition and a lower abundance of the Proteobacteria Mucositis lesions: an increased abundance of *Peptostreptococcus, Lactobacillus*, and *Mycoplasma*
Osakabe et al. [42]	Patients with hematological malignancies (*n* = 19)	Myeloablative or reduced-intensity conditioning + HSCT	Bilateral buccal mucosa, tongue, and palate samples/mass spectrometer	Post-HSCT, a decrease in *Streptococcus spp*. and an increase in coagulase-negative *staphylococci* were observed OM was significantly associated with an increase in *Candida spp*. and detection of *Enterococcus spp*.
Zhu et al. [43]	Patients with nasopharyngeal carcinoma (*n* = 41) Healthy controls (*n* = 49)	Radiotherapy/ chemoradiotherapy	Retropharyngeal mucosa or lesion swabs/16S rRNA gene sequencing	Radiotherapy caused progressive alterations in the bacterial community structure with an increase in the relative abundance of Gram-negative bacteria Patients who developed severe OM had a significantly lower alpha diversity and higher *Actinobacillus* during the erythema phase
Hou et al. [44]	Patients with nasopharyngeal carcinoma (*n* = 19)	Radiotherapy	Oropharyngeal mucosa swabs/16S rRNA gene sequencing	No change in bacterial alpha diversity during treatment 20 genera were positively associated and 10 negatively associated with radiation dose The abundance of *Prevotella, Porphyromonas, Fusobacterium*, and *Treponema* showed dynamic variations during radiotherapy, with peak abundance at severe OM onset
Vesty et al. [45]	Patients with HNC (*n* = 19)	Radiotherapy	Saliva and buccal mucosa swabs/16S rRNA gene sequencing	*Saliva:* *Parviomonas micra, Capnocytophaga leadbetteri, Olsenella uli, Neisseria mucosa*, and *Tannerella forsythia* were enriched in patients with ≥ grade 2 OM The abundance of *Bacteroidales G2, Capnocytophaga, Eikenella, Mycoplasma, Sneathia, periopathogenic Porphyromonas*, and *Tannerella* genera were positively correlated with ≥ grade 2 OM *Buccal mucosa:* Increased relative abundance of *Fusobacterium, Bacteroidales G2*, and *Sneathia* in ≥ grade 2 OM The abundance of *Fusobacterium, Porphyromonas, Haemophilus, Eikenella*, and *Tannerella* are associated with OM risk
Hong et al. [41]	Adult patients with cancer (*n =* 49) Healthy control (*n* = 30)	Chemotherapy (5-fluorouracil or doxorubicin)	Saliva and mucosal swabs/16S rRNA gene sequencing	Oral bacteria disruption was strongly associated with OM severity OM was associated with depletion of commensal bacteria belonging to *Streptococcus, Actinomyces Veillonella, Granulicatella*, and *Gemella* genera and enrichment of *Fusobacterium nucleatum* and *Prevotella oris*. OM-enriched *F. nucleatum* displayed pro-inflammatory and pro-apoptotic capacity
Laheij et al. [46]	Patients with multiple myeloma (*n =* 51)	High dose melphalan + autologous HSCT	Oral rinse samples/16S rRNA gene sequencing	Significant alteration in oral microbiota post- autoSCT which recovered within three months More pronounced changes in oral microbial diversity in patients who developed ulcerative OM Distinctive pre-autoSCT taxa discriminate between patients who developed OM and those who did not Pre-autoSCT, patients who developed OM had increased abundance of in *Veillonella, Enterococcus faecalis, Streptococcus, Staphylococcus spp., Fusobacterium, Prevotella oris*, and *Prevotella veroralis*, and reduced abundance of *Actinomyces graevenitzii* and *Streptococcus constellatus* Patients who did not develop ulcerative OM had a more resilient microbial community
Mougeot et al. [47]	Patient with hematological cancers (*n* = 22)	Conditioning regimens + HSCT	Saliva and buccal mucosa, tongue, and supragingival plaque swabs/16S rRNA gene sequencing	Patients with score 2 OM had increased abundance of *Gammaproteobacteria* (*Escherichia-Shigella* genus) and decreased abundance of *Haemophilus parainfluenza* *Veillonella* enriched in patients with score 1-2 OM
Reyes-Gibby et al. [48]	Patients with HNSCC (*n =* 66)	Chemotherapy/ radiotherapy/ chemoradiotherapy	Buccal mucosa swabs/16S rRNA gene sequencing	At baseline: a higher abundance of *Cardiobacterium* and *Granulicatella* was associated with early onset of severe OM (grade 3) Immediately before OM development: an increased abundance of *Prevotella* and *Fusobacterium*, and decreased abundance of *Streptococcus* were associated with the early onset of severe OM Immediately before severe OM development: an increased abundance of *Megasphaera* and *Cardiobacterium* was associated with the early onset of severe OM
Shouval et al. [49]	Patients with hematological conditions (*n* = 184) Healthy controls (*n* = 19)	High intensity/ myeloablative conditioning + allogeneic HSCT	Saliva/16S rRNA gene sequencing	HSCT was associated with a decrease in oral alpha diversity Pre-HSCT: an increased abundance of *Kingella* and *Atopobium* correlated to a higher risk of developing severe OM (grade 3-4) Post-HSCT: *Methylobacterium spp*. were enriched in patients with severe OM, while *Treponema* and *TG5* were increased in grade 0-1 OM A more pronounced change in the salivary microbial diversity and metabolites post-HSCT in those developed grade 3-4 OM
Takahashi et al. [50]	Patients with hematological malignancies (*n* = 19) Healthy controls (*n* = 3)	Cyclophosphamide + total body irradiation OR fludarabine and melphalan + HSCT	Tongue, buccal mucosa, and teeth swabs/16S rRNA gene-based terminal restriction fragment length polymorphism (T-RFLP)	Patients with severe OM had larger changes in the oral bacterial community post-HSCT than patients with mild OM Faster recovery of the microbial diversity and abundance in patients with mild/moderate OM compared to patients with severe OM

Most of the present research has focused on the association between oral microbiota and OM; however, the causal relationship remains poorly understood. Only one study has been conducted and demonstrated that germ-free mice treated with chemotherapy had less oral epithelial tissue injury and lower levels of pro-inflammatory cytokines and matrix metalloproteinases in the tongue tissues compared to specific pathogen-free mice [51]. Although the authors suggested that these outcomes are mediated by the oral microbiota, this does not exclude the impact of the gut microbiota as germ-free mice are completely free of all microbes. Overall, despite limited research, current evidence suggests that oral microbiota may contribute to OM through the regulation of oral innate immune pathways including NF-κB and toll-like receptors (TLRs) [22]. Microbiota-derived molecules like lipopolysaccharides can interact with TLRs in infiltrating immune cells leading to the further activation of NF-κB and, therefore, exacerbating inflammatory signals [21]. Further, the oral microbiota could influence OM healing phase by regulating the rate of mucosal recovery and restoration. It has been demonstrated that co-culturing the oral microbiota biofilms and epithelial cell layer alters its wound healing capacity [52]. Moreover, oral pathobiont associated with OM e.g., *Porphyromonas gingivalis* (*P. gingivalis)* has been found to inhibit cell migration in an *in vitro* assay of human buccal epithelial cells, suggesting the oral microbiota could contribute to the epithelial wound healing process [53, 54].

### OM-Associated Intestinal Dysfunction

**Figure 1:**
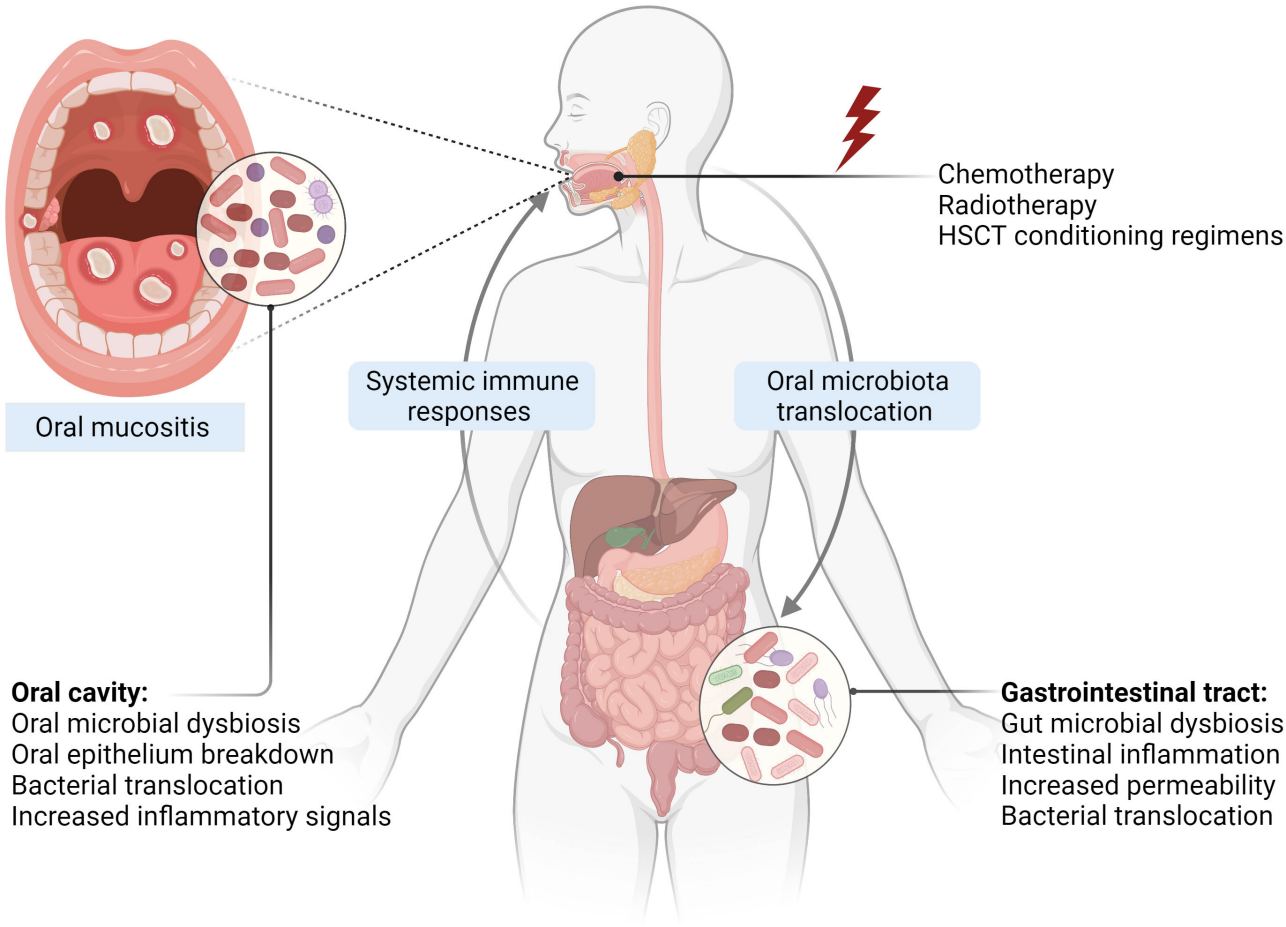
Oral-gut microbiome axis in the development of OM. Exposure to cytotoxic cancer treatments causes direct tissue injury and subsequent inflammatory responses leading to epithelial damage. Changes in the oral environment result in oral microbiota dysbiosis, which can cross through the damaged and ulcerated mucosa, interacting with immune cells and enhancing inflammatory responses. Intestinal pathological changes including gut microbiota dysbiosis, caused by anticancer agents and oral microbiota translocation into the gut, disrupt intestinal homeostasis and facilitate bacterial translocation into circulation and activation of systemic immune responses, which in turn aggravate OM severity (Created with Biorender.com).

It is well-documented that systemic chemotherapy and HSCT myeloablative regimes cause significant gastrointestinal toxicities characterized by gastrointestinal mucositis, diarrhea, nausea, vomiting, and abdominal pain [55]. These toxicities are often associated with major gastrointestinal pathological changes including gut microbial dysbiosis, disruption of barrier functions, and intestinal inflammation [13, 25]. While these are expected consequences in patients receiving systemic therapies, local radiotherapy to the head and neck could also cause intestinal inflammation and disrupt intestinal barriers. For instance, Fernández-Gil et al. demonstrated that irradiation of the rat oral cavity was associated with intestinal damage, oxidative stress, and reduction in intestinal tight junction protein, Zonula occludens-1 [56]. Gastrointestinal toxicity characterized by disruption of intestinal barriers can lead to increased translocation of bacterial endotoxins into the circulation, activation of systemic inflammation, and eventually aggravating tissue injury in other parts of the body such as the brain [57, 58], liver [59], and heart [60]. Similarly, these pathological changes could enhance the severity of OM by enhancing systemic inflammatory responses; however, this is yet to be investigated. Nevertheless, reduced intestinal inflammation and increased expression of tight junction proteins were associated with lower severity of radiation-induced OM in a rat model suggesting that intestinal homeostasis is a potential target for alleviating OM [56]. Together, intestinal pathologies during cancer therapies may contribute to OM development and severity through activating systemic inflammation (Figure 1), and hence further research is warranted.

### Bottom-Up: Gut Microbiota Dysbiosis and Its Impact on OM

The gut microbiota refers to trillions of microorganisms found along the gastrointestinal tract [61]. Our understanding of these microbes has evolved enormously, and it is now well established that the gut microbiota controls the development and modulation of several host physiological processes including intestinal homeostasis, immune responses, and host metabolism [62]. As such, perturbations in the gut microbiota have been implicated in several intestinal and extraintestinal conditions at distant sites [63]. It has been widely demonstrated that the gut microbiota is disrupted in patients at high risk of OM, including those undergoing systemic chemotherapy or HSCT [64, 65]. While HNC local radiotherapy is not expected to cause a significant change in the gut microbiota, chemoradiotherapy regimens may lead to gut microbial dysbiosis in patients with HNC. Currently, only one study has investigated the impact of chemoradiotherapy on the gut microbiota of patients with oropharyngeal cancer and reported no changes in the gut microbiota post chemoradiotherapy [66]. However, this was only evaluated in a small sample size (*N* = 22) with limited resolution; hence further research is required.

Since the gut microbiota plays a critical role in modulating systemic immune and inflammatory responses, it may influence the development and/or severity of OM [34, 67]. However, the current evidence supporting this is limited. As discussed earlier, germ-free mice (lacking both oral and gut microbiota) are less susceptible to oral injury and inflammation following chemotherapy [51]. Similarly, we have shown that treating rats with broad-spectrum antibiotics in drinking water, to deplete the gut microbiota, decreased radiation-induced OM severity by reducing the inflammatory cytokines in tongue tissues [68]. Although it is difficult to dissect whether these findings are due to changes in the oral or gut microbiota, the immunomodulatory capacity of the gut microbiota is undoubtedly larger than that of the oral microbiota. As such, it is likely that the gut microbiota plays an important role in OM pathobiology. In fact, this concept is supported by more recent evidence which used a more targeted approach to deplete the gut microbiota using intragastric antibiotics. Mice exposed to antibiotics had reduced epithelial damage and immune cell infiltration in the tongue after irradiation, indicating that gut microbiota is implicated in OM development [69]. Minimal effects of intragastric antibiotics on the oral microbiota were reported suggesting that the protective effect is mainly mediated by the gut microbiota depletion independent of the oral microbiota [69].

Mechanistically, it is well-known that gut microbiota plays a pivotal role in maintaining intestinal homeostasis and enhancing intestinal barrier integrity [62]. Therefore, the disruption of the steady-state balance of the gut microbiota could indirectly influence OM by further aggravating the disruption of intestinal integrity caused by anticancer agents and hence activating systemic immune responses [70]. A recent study demonstrated that the restoration of the gut microbiota using ingested probiotics reduced the severity of OM in patients with nasopharyngeal cancer receiving chemoradiotherapy and in a rat model of radiation-induced OM through reducing of OM-associated inflammation [71]. Collectively, growing research indicates that gut microbiota could play a role in OM pathology (Figure 1); however, further research in this field is needed.

### Top-Down: Interaction Between the Oral and Gut Microbiota During OM

The oral and gut microbiota are composed of distinctive microbial load and taxa. However, the interconnected nature of the alimentary tract provides a potential route of oral bacteria transfer into the distal digestive tract. It has been hypothesized that oral microbiota can translocate into the gut through either the enteral (gastrointestinal tract) or the hematological route (blood) [72, 73]. Current evidence suggests that more than half of oral microbes are subjected to oral-gut translocation even in healthy individuals [74]. However, more pronounced ectopic displacement occurs under pathological conditions such as periodontitis and severe systemic inflammatory disorders [75]. Oral microbiota colonization of the gut, also known as the oralization of the gut microbiota, has been linked to several conditions including liver cirrhosis [76] and colorectal cancer [77]. The translocation of oral pathobionts could result in gut microbial dysbiosis and potentially disrupt intestinal immune homeostasis, hence affecting gastrointestinal [78] and systemic inflammatory diseases [79]. For instance, the administration of *P. gingivalis* was found to cause a significant gut microbiota dysbiosis, reduce the expression of intestinal tight junction proteins and increase the risk of endotoxemia [80, 81]. Collectively, oral bacteria translocation is increased in pathological conditions and could cause gut microbiota dysbiosis and disruption of intestinal homeostasis.

Oral microbiota translocation during OM is yet to be investigated. Nevertheless, an increase in oral bacteria in the gut has been reported following cancer treatments [82, 83]. It has been demonstrated that oral Firmicutes (*Veillonella parvula* and *Solobacterium moorei*) and Actinobacteria (*Rothia mucilaginosa*) are detected in the stool of patients undergoing HSCT and are associated with the severity of acute graft-vs.-host disease [82, 83]. Since OM is associated with major changes in the oral environment and oral microbial community, translocation of dysbiotic oral bacteria into the gut is likely to occur. This in turn could contribute to pathological changes in the gut and activation of systemic immune responses and hence negatively affect OM (Figure 1). As such, further research investigating oral microbial translocation in patients at risk of OM and whether that has any implications in OM pathogenies is warranted.

## Targeting The Oral-Gut Microbiota Axis in OM

Since the recognition of the potential role of oral bacteria in the pathogenesis of OM, multiple attempts to use antiseptic and antimicrobial agents to treat or prevent OM in patients undergoing cancer treatments have been made with limited success [84–87]. The lack of benefit seen in these studies could be due to the use of non-targeted antimicrobial agents. Further, the use of antibiotics could disrupt the oral microbial ecosystem affecting both commensal and pathobiont microbes and hence may have overall detrimental effects on OM. As such, the use of alternative methods such as probiotics has been explored [21, 88, 89]. In a recent systematic review, which included five clinical trials, probiotics reduced the risk of all OM grades with a more significant result for grade ≥3 [90]. Probiotics could be used to manipulate oral and gut microbiota to improve both oral and intestinal homeostasis. For instance, administration of probiotic feed containing *Bacillus subtilis, Bifidobacterium bifidum, Enterococcus faecium, and Lactobacilllus acidophilus*, has been shown to enhance OM regression and reduce both oral and intestinal inflammation and intestinal villus-related damage in a rat model of chemotherapy-induced OM [91]. Probiotics are a safe method for modulating the microbiota; however, the risk of infections should be taken into consideration, especially in immunocompromised patients. Although, it should be appreciated that a damaged microbiota is predictive of infection in immunocompromised patients, and as such, probiotics may counterintuitively serve to reduce infection risk.

Another way to modulate gut microbiota is through diet. Given that reduction of oral intake is one of the main OM complications, changes in dietary habits are likely to have a significant impact on the gut microbiota. Andersen et al. demonstrated that reduced oral intake post hematopoietic progenitor cell transplantation was associated with a shift in the microbial composition with a lower gut microbial diversity and lower abundance of *Blautia* and Faecalibacterium *prausnitzii* [92]. Furthermore, compared to parenteral nutrition, enteral nutrition was associated higher abundance of short-chain fatty acids-producing *Faecalibacterium* and *Ruminococcus bromii* [92] and faster recovery of the gut microbiota structure [93]. Therefore, further research is needed to determine the best nutritional support that enriches the oral and gut microbiota symbiosis of patients suffering from OM.

Fecal microbiota transplant (FMT) and more recently oral microbiota transplant (OMT) are also possible ways to restore microbial symbiosis. While FMT is a more well-established procedure, it is yet to be investigated for mitigation of OM. Further, only one study has demonstrated that OMT from healthy mice into irradiated mice was able to reduce OM-associated epithelial injury and oral and systemic inflammation by mitigating irradiation-induced alteration in both oral and gut microbiota [69]. Further research is warranted for both FMT and OMT as they hold significant potential as do other emerging strategies such as photobiomodulation [94, 95].

## Conclusion

Cancer treatment-induced OM remains a major complication with significant personal, clinical, and economic burdens. Growing evidence indicates that the oral microbiota is altered following cancer treatment and may be involved in OM pathogenesis. Further, there is mounting evidence for the role of the gut microbiota contributing to OM pathogenesis through the regulation of systemic immune responses. Moreover, intestinal dysfunction caused by cancer treatment or oralization of gut microbiota could exacerbate the severity of OM. Further research is warranted to further investigate these oral-gut microbiome axis pathways and identify the best targeting intervention to prevent or reduce the severity of OM.

## Author Contributions

GA-Q conceptualized, wrote, and contributed to editing the manuscript. HW, YV, and JB contributed to drafting and revising the manuscript. All authors contributed to the article and approved the submitted version.

## Conflict of Interest

The authors declare that the research was conducted in the absence of any commercial or financial relationships that could be construed as a potential conflict of interest.

## Publisher's Note

All claims expressed in this article are solely those of the authors and do not necessarily represent those of their affiliated organizations, or those of the publisher, the editors and the reviewers. Any product that may be evaluated in this article, or claim that may be made by its manufacturer, is not guaranteed or endorsed by the publisher.
